# Supplementary figures and images for: Creative Action at a Distance: A Conceptual Framework for Embodied Performance With Robotic Actors
Source: Front Robot AI. 2021 Apr 30;8:662182. doi: 10.3389/frobt.2021.662182 (PMC8120109; doi:10.3389/frobt.2021.662182)

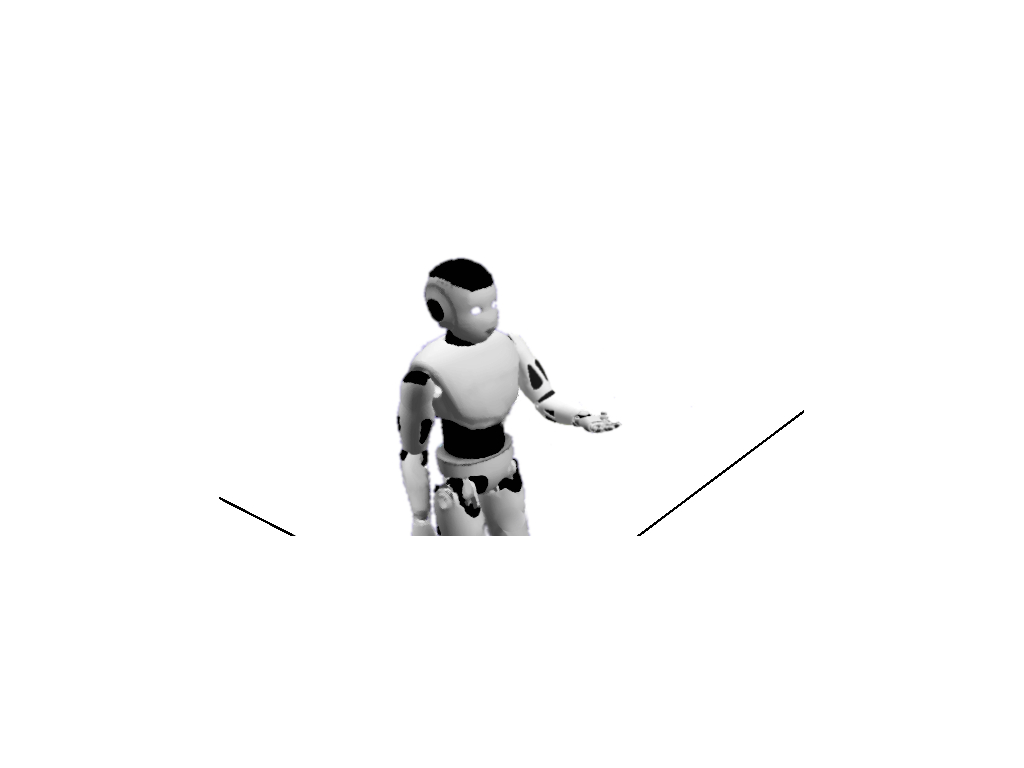

Supplement: Supplementary file 1 [file Image3.JPEG]

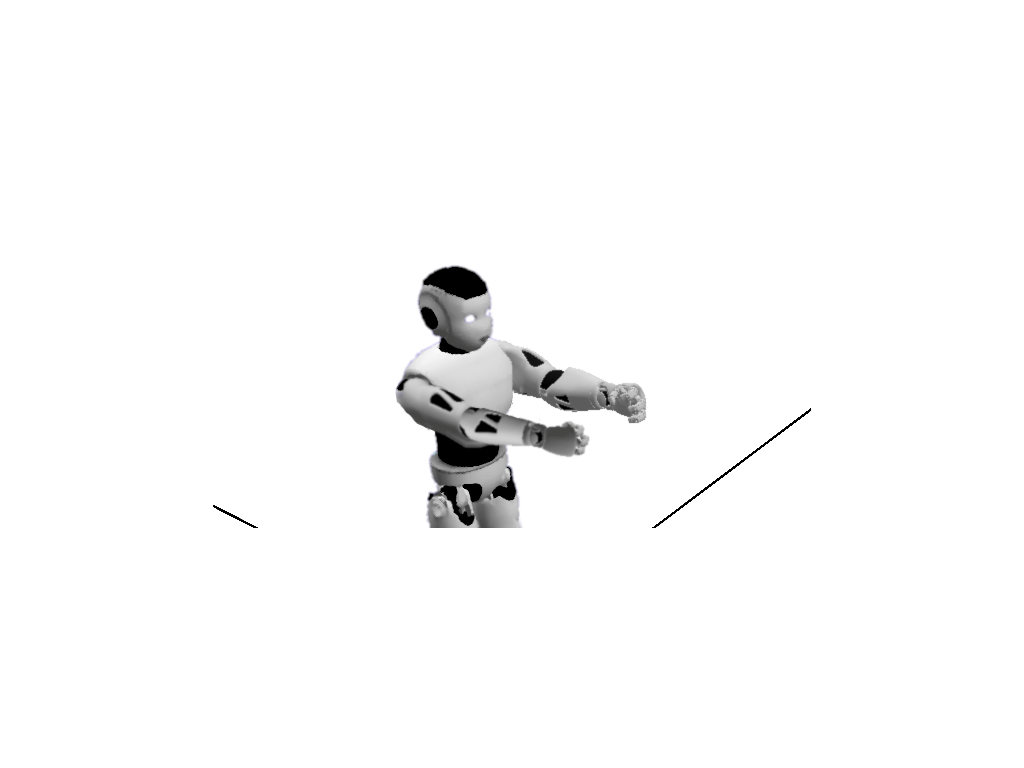

Supplement: Supplementary file 2 [file Image1.JPEG]

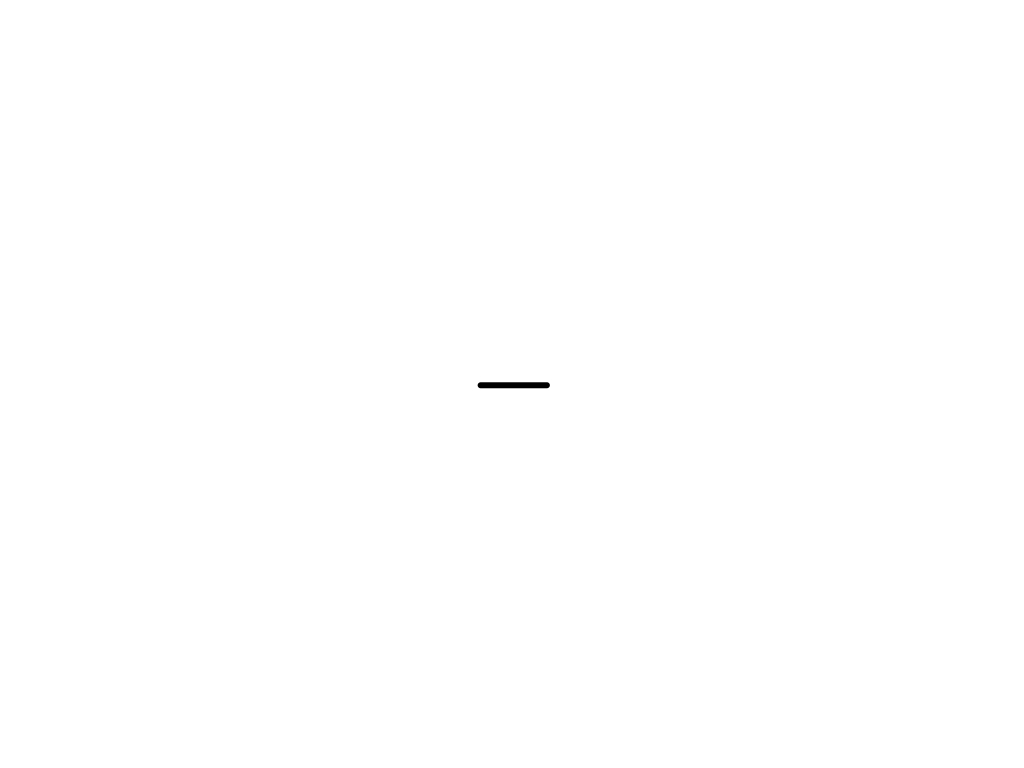

Supplement: Supplementary file 3 [file Image4.JPEG]

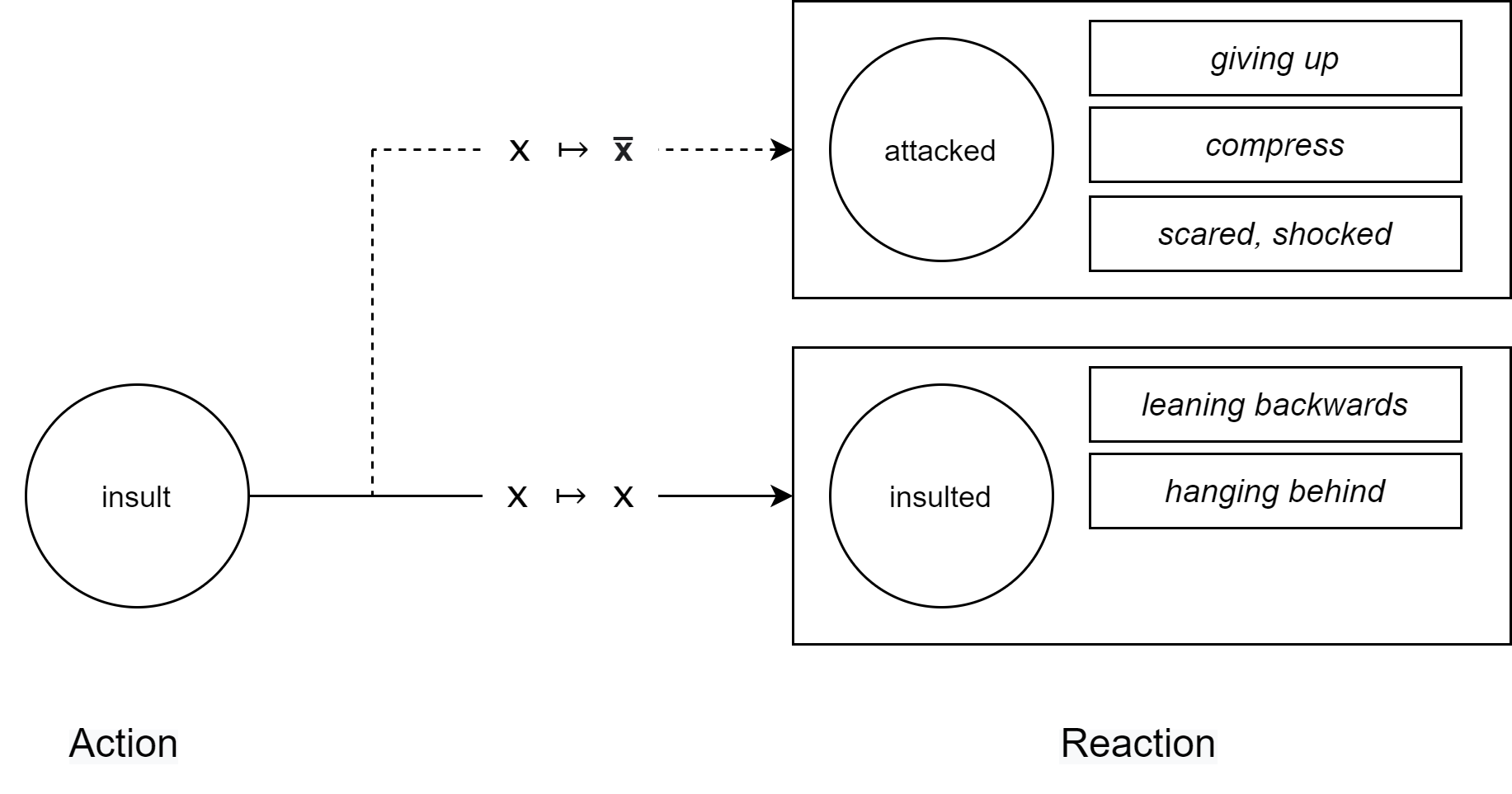

Supplement: Supplementary file 4 [file DataSheet1.ZIP › Frontiers_Creativity_Robotics_2020_Revised/graphics/actionflow.png]

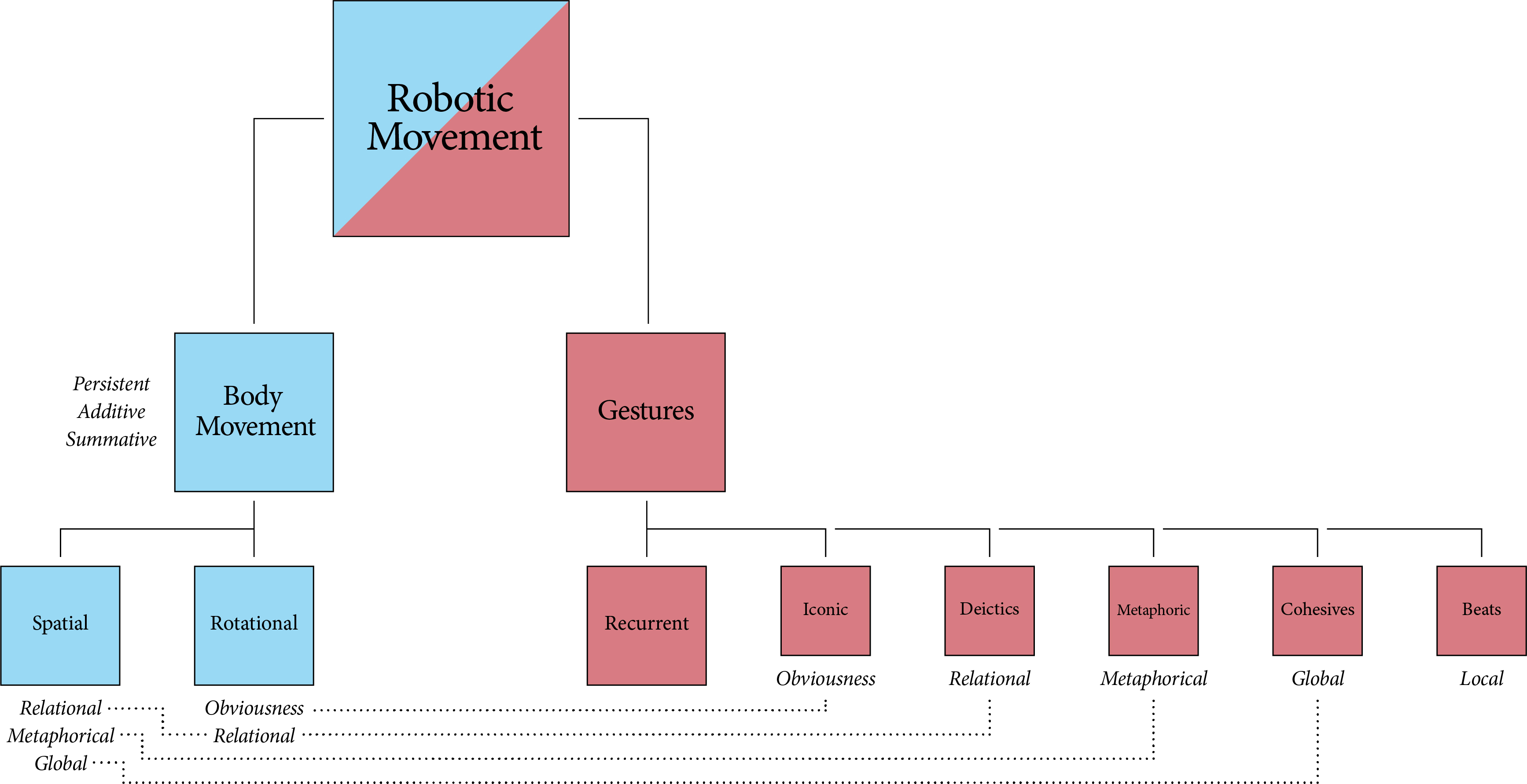

Supplement: Supplementary file 4 [file DataSheet1.ZIP › Frontiers_Creativity_Robotics_2020_Revised/graphics/figure_01.jpg]

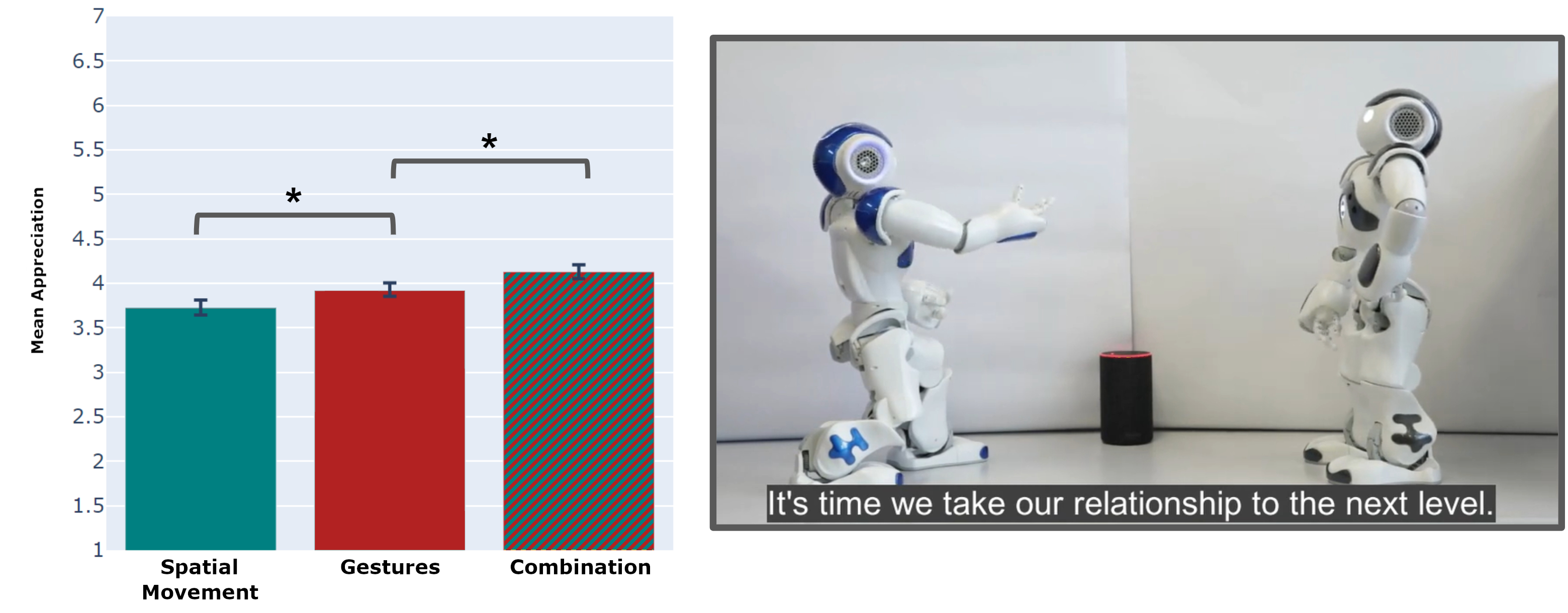

Supplement: Supplementary file 4 [file DataSheet1.ZIP › Frontiers_Creativity_Robotics_2020_Revised/graphics/graph1.jpg]

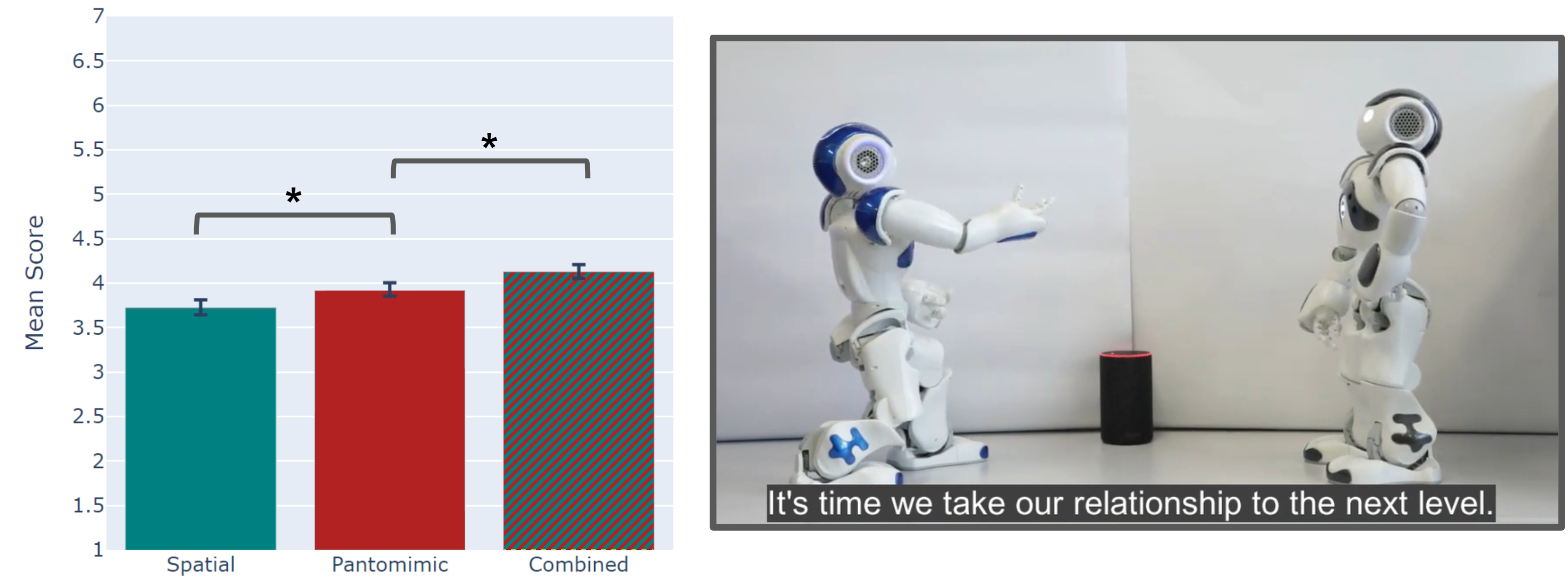

Supplement: Supplementary file 4 [file DataSheet1.ZIP › Frontiers_Creativity_Robotics_2020_Revised/graphics/graph2.jpg]

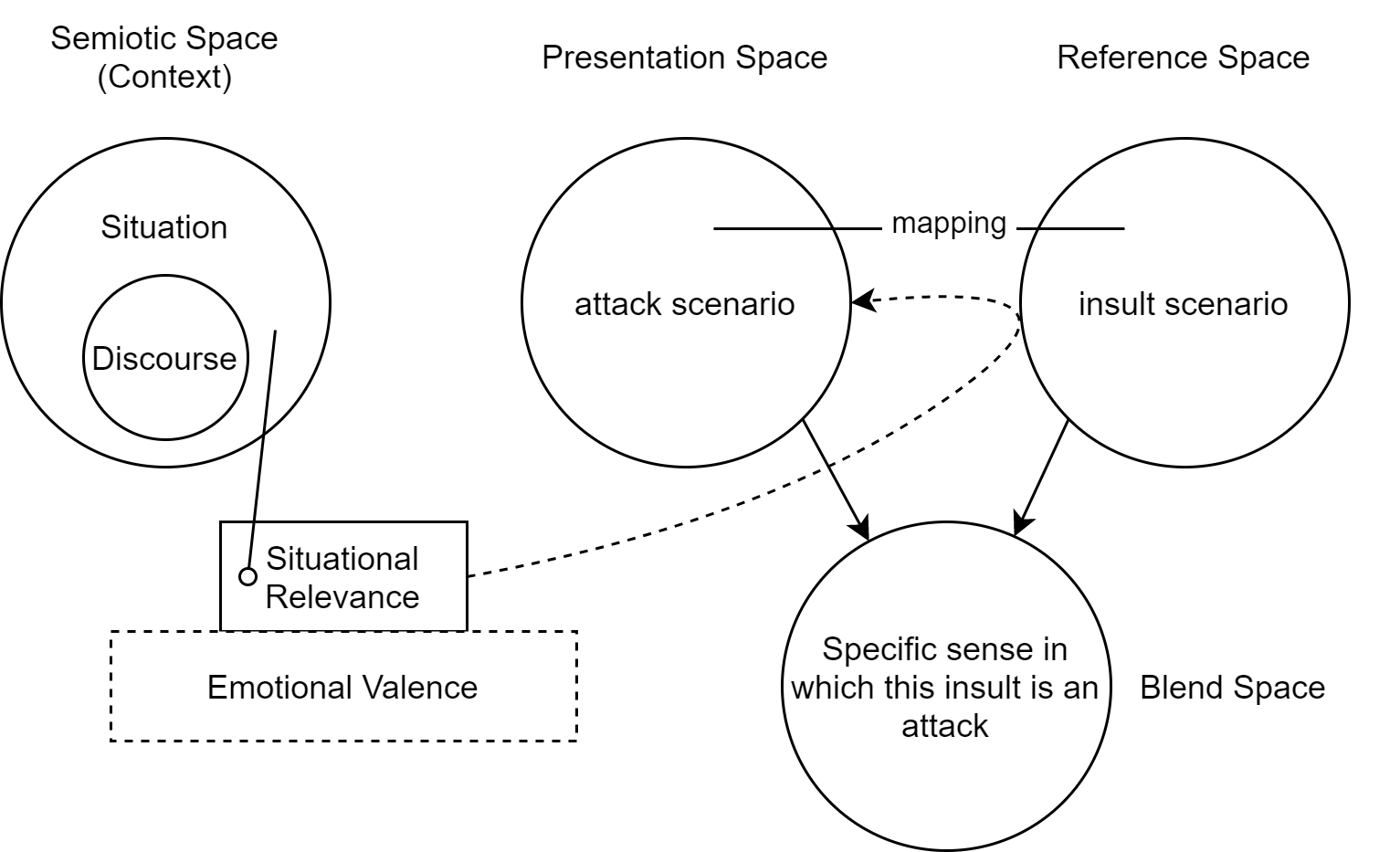

Supplement: Supplementary file 4 [file DataSheet1.ZIP › Frontiers_Creativity_Robotics_2020_Revised/graphics/mapping.png]

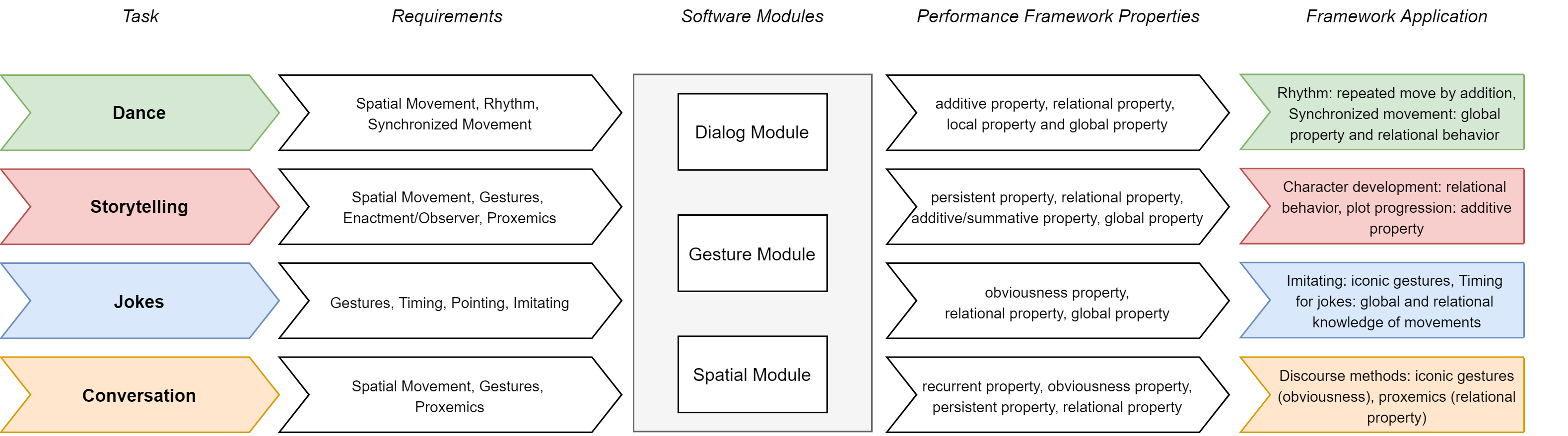

Supplement: Supplementary file 4 [file DataSheet1.ZIP › Frontiers_Creativity_Robotics_2020_Revised/graphics/model_04.png]

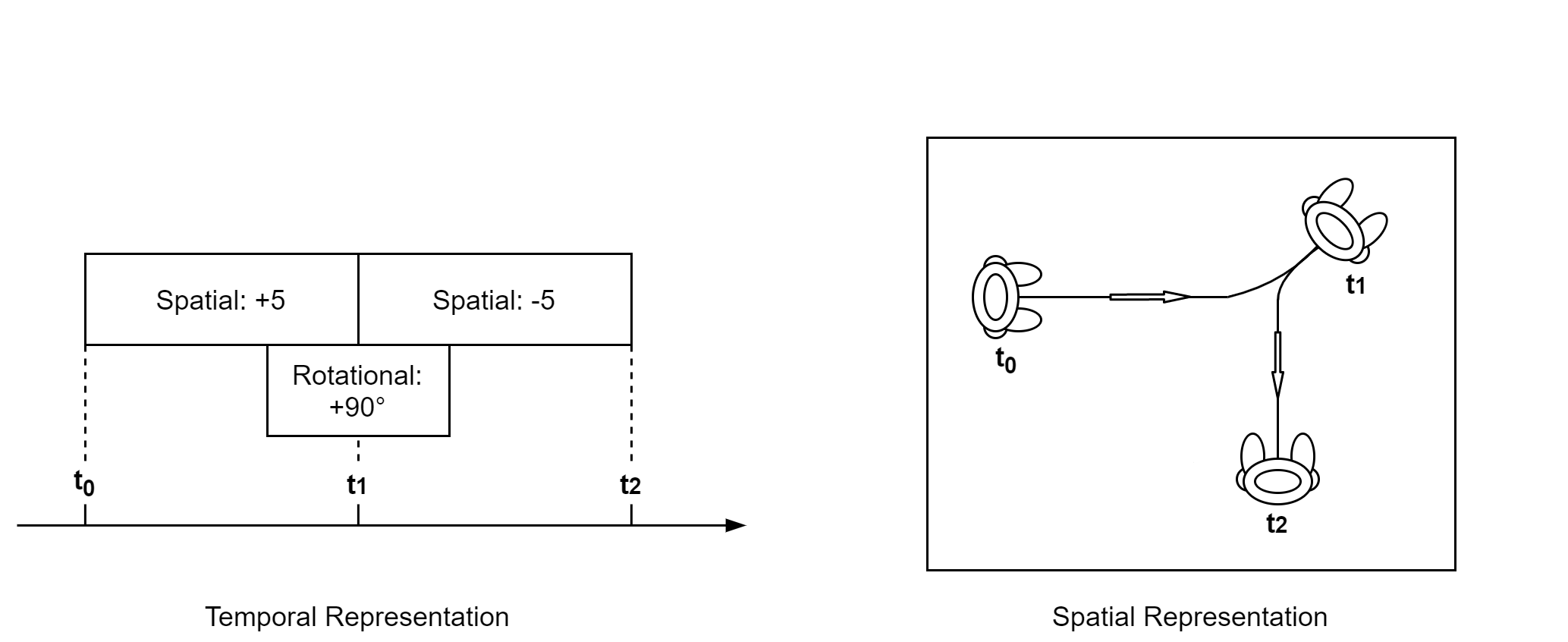

Supplement: Supplementary file 4 [file DataSheet1.ZIP › Frontiers_Creativity_Robotics_2020_Revised/graphics/moves.png]

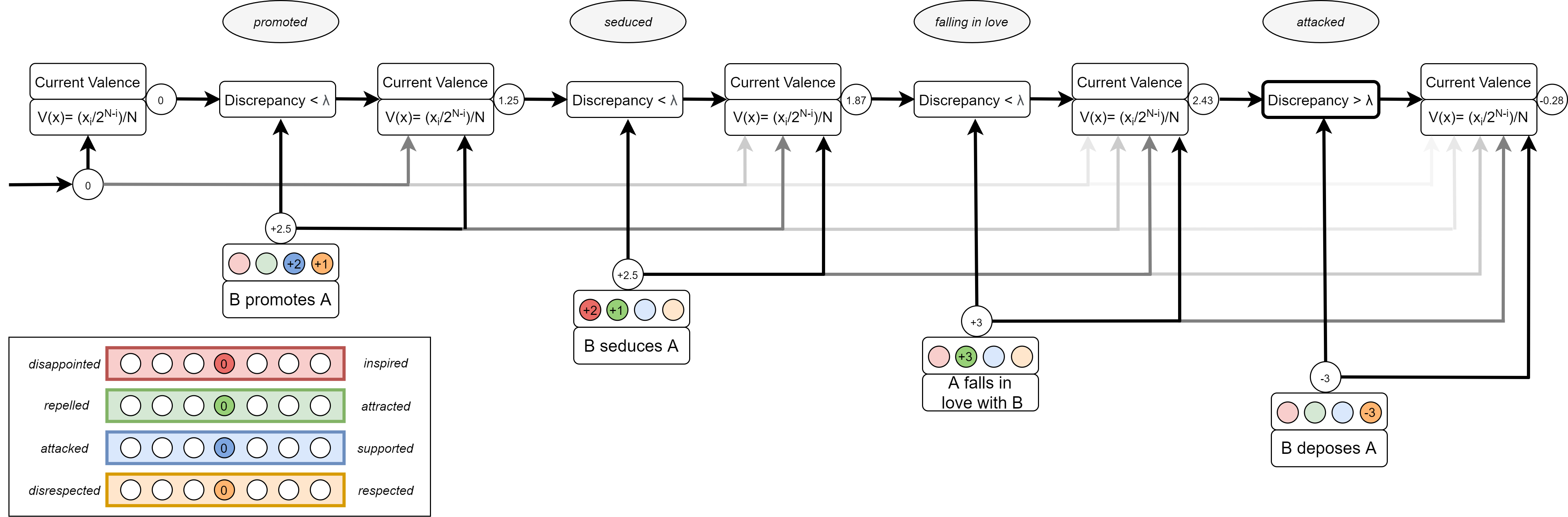

Supplement: Supplementary file 4 [file DataSheet1.ZIP › Frontiers_Creativity_Robotics_2020_Revised/graphics/plot3.png]

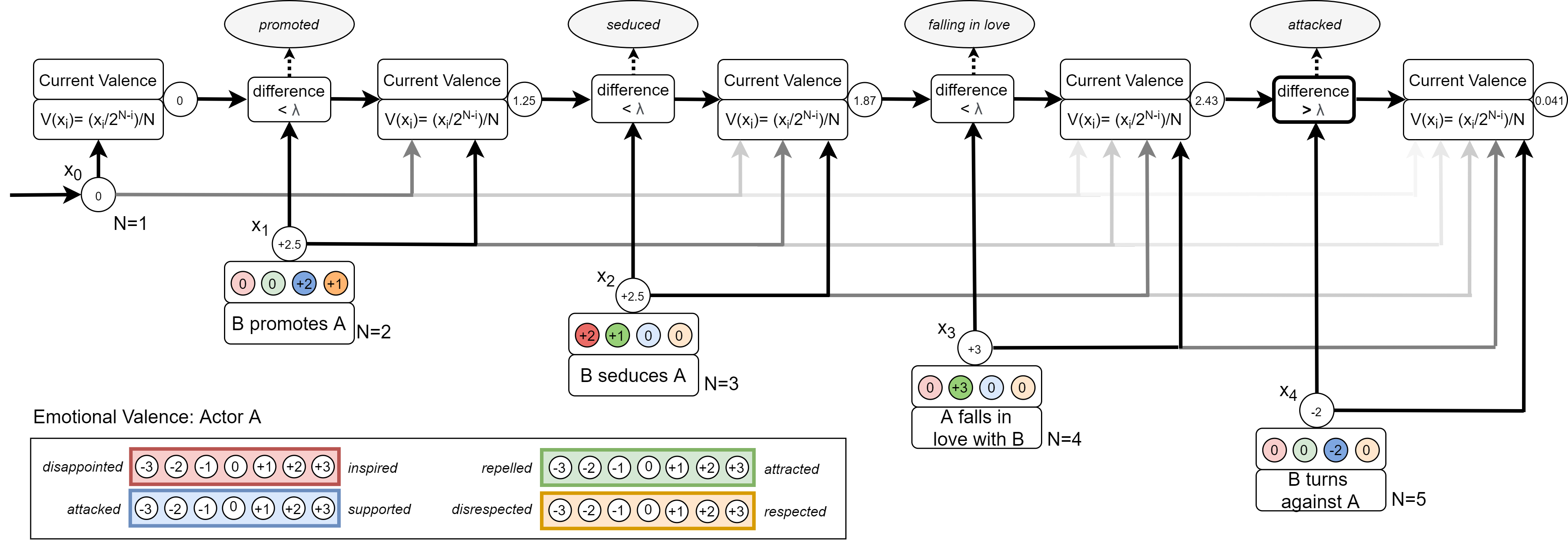

Supplement: Supplementary file 4 [file DataSheet1.ZIP › Frontiers_Creativity_Robotics_2020_Revised/graphics/plot4.png]

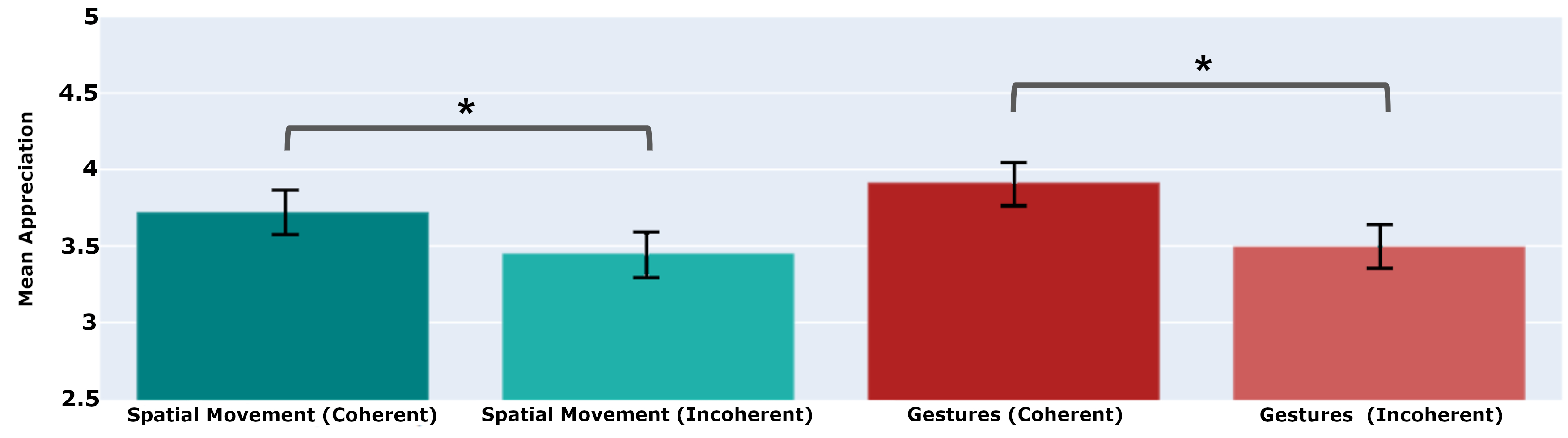

Supplement: Supplementary file 4 [file DataSheet1.ZIP › Frontiers_Creativity_Robotics_2020_Revised/graphics/results01.jpg]

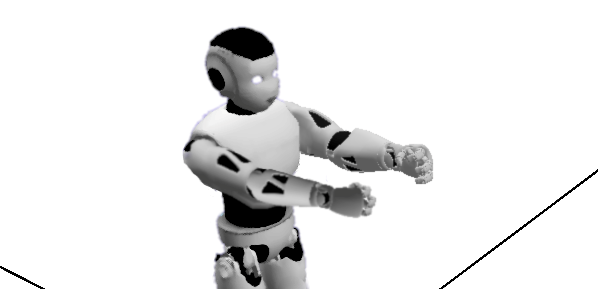

Supplement: Supplementary file 4 [file DataSheet1.ZIP › Frontiers_Creativity_Robotics_2020_Revised/graphics/robot01_driveTorso.png]

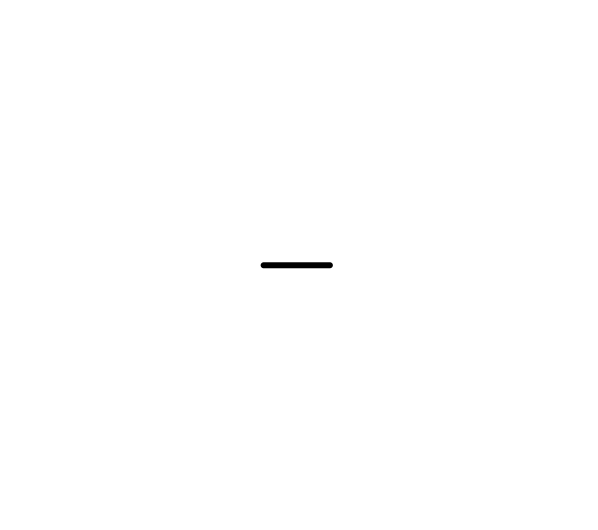

Supplement: Supplementary file 4 [file DataSheet1.ZIP › Frontiers_Creativity_Robotics_2020_Revised/graphics/robot01_none.png]

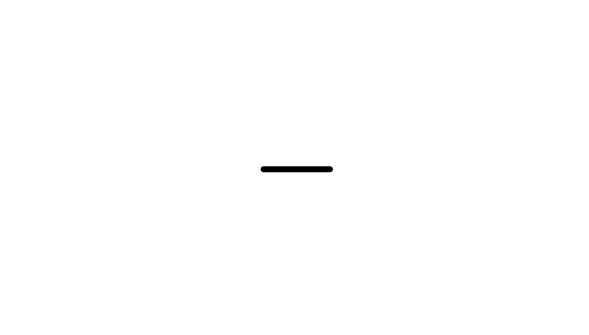

Supplement: Supplementary file 4 [file DataSheet1.ZIP › Frontiers_Creativity_Robotics_2020_Revised/graphics/robot01_noneSmall.png]

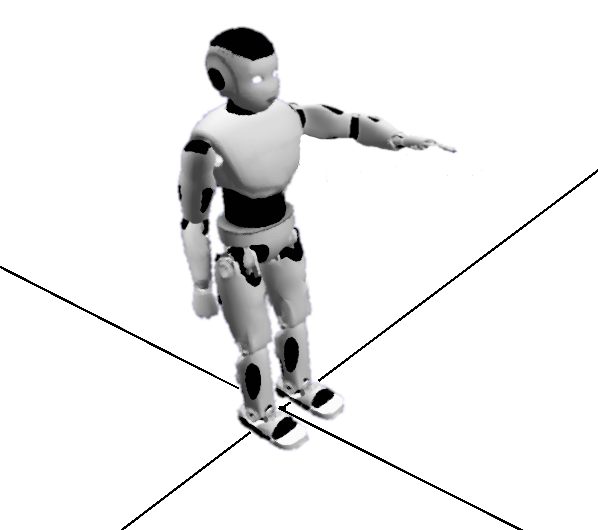

Supplement: Supplementary file 4 [file DataSheet1.ZIP › Frontiers_Creativity_Robotics_2020_Revised/graphics/robot01_point.png]

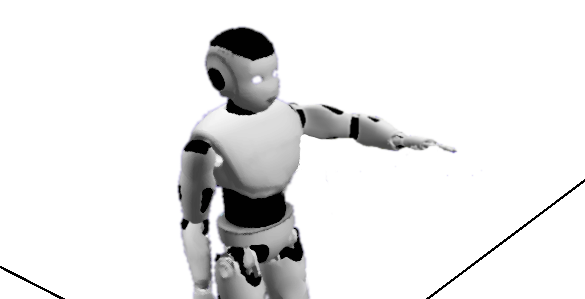

Supplement: Supplementary file 4 [file DataSheet1.ZIP › Frontiers_Creativity_Robotics_2020_Revised/graphics/robot01_pointTorso.png]

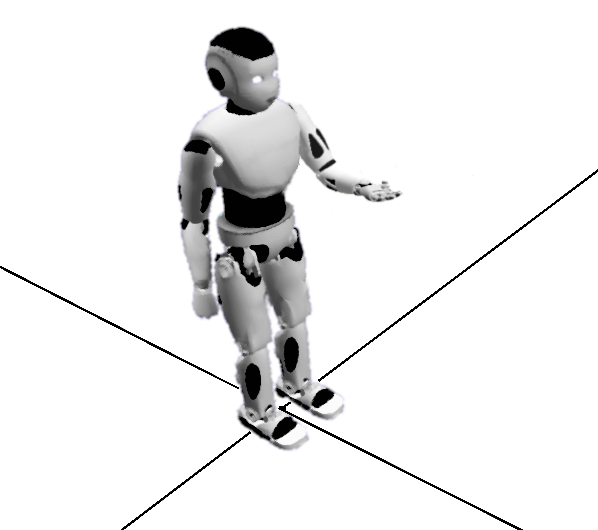

Supplement: Supplementary file 4 [file DataSheet1.ZIP › Frontiers_Creativity_Robotics_2020_Revised/graphics/robot01_puoh.png]

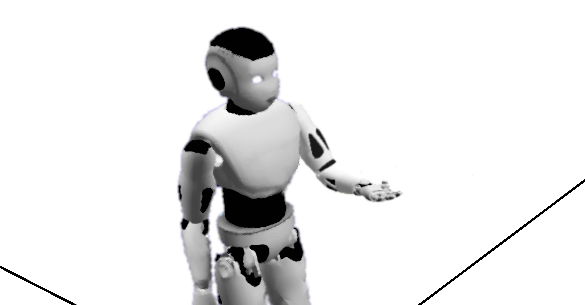

Supplement: Supplementary file 4 [file DataSheet1.ZIP › Frontiers_Creativity_Robotics_2020_Revised/graphics/robot01_puohTorso.png]

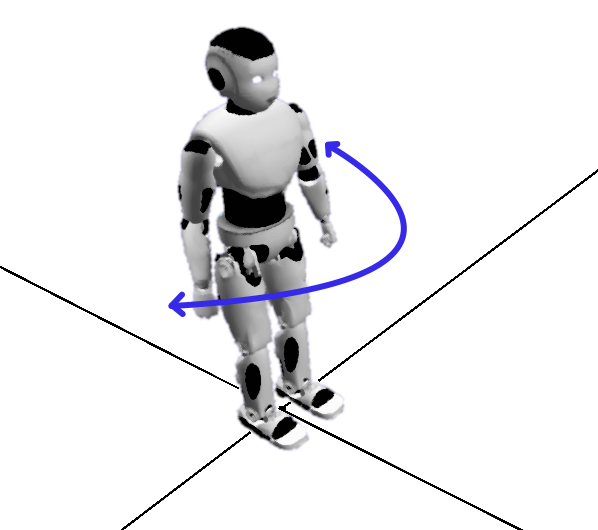

Supplement: Supplementary file 4 [file DataSheet1.ZIP › Frontiers_Creativity_Robotics_2020_Revised/graphics/robot01_rot.png]

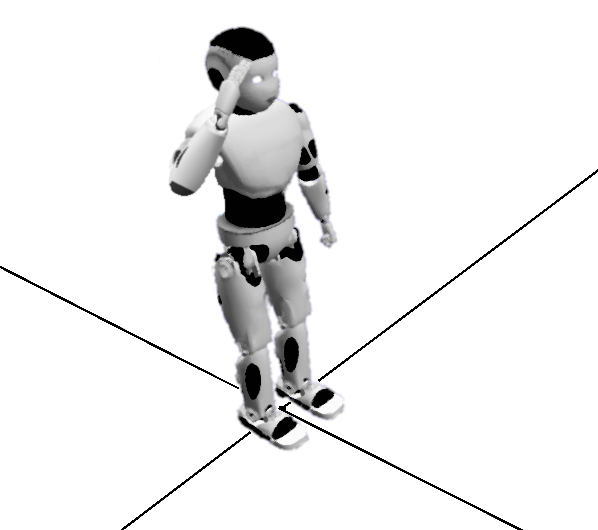

Supplement: Supplementary file 4 [file DataSheet1.ZIP › Frontiers_Creativity_Robotics_2020_Revised/graphics/robot01_salute.png]

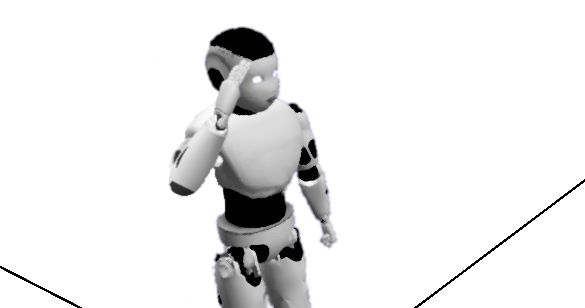

Supplement: Supplementary file 4 [file DataSheet1.ZIP › Frontiers_Creativity_Robotics_2020_Revised/graphics/robot01_saluteTorso.png]

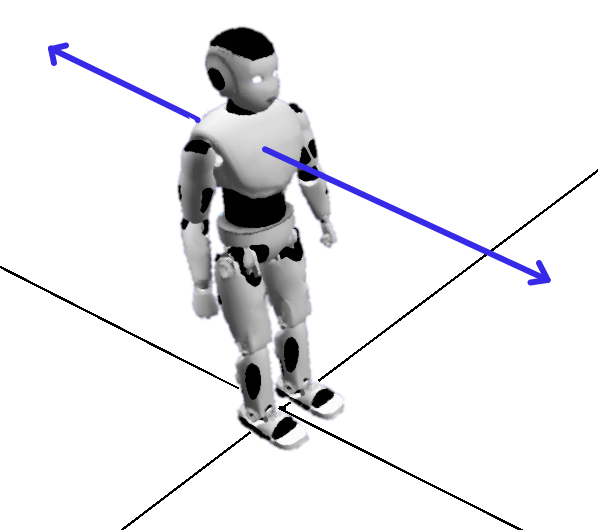

Supplement: Supplementary file 4 [file DataSheet1.ZIP › Frontiers_Creativity_Robotics_2020_Revised/graphics/robot01_spat.png]

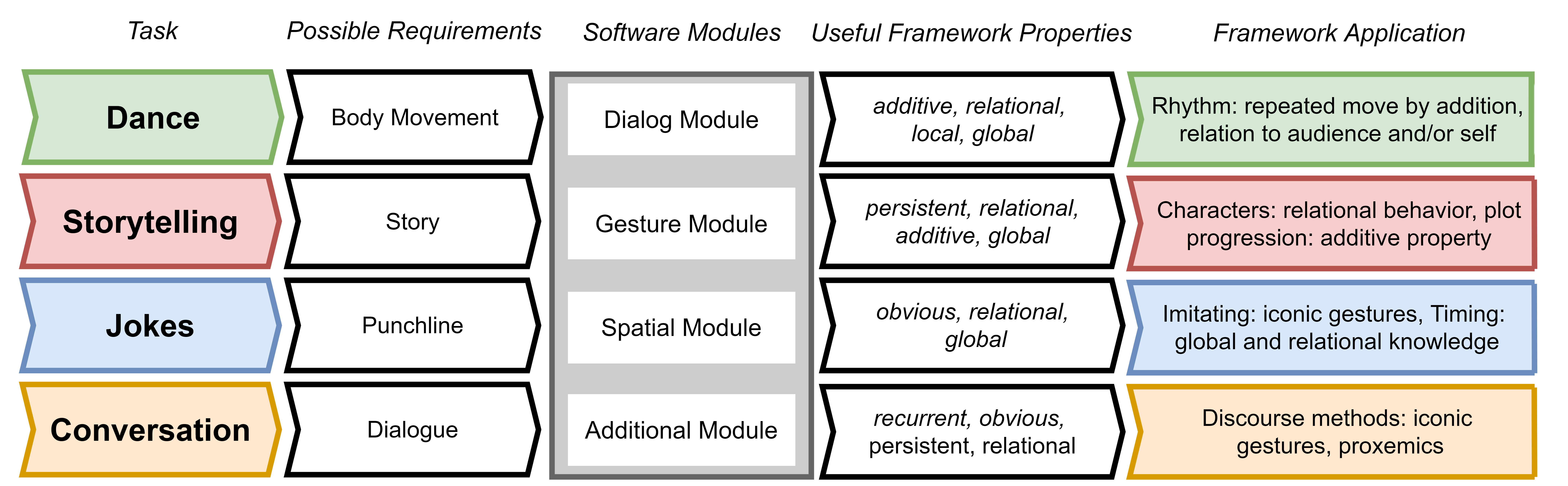

Supplement: Supplementary file 4 [file DataSheet1.ZIP › Frontiers_Creativity_Robotics_2020_Revised/graphics/tasks.png]

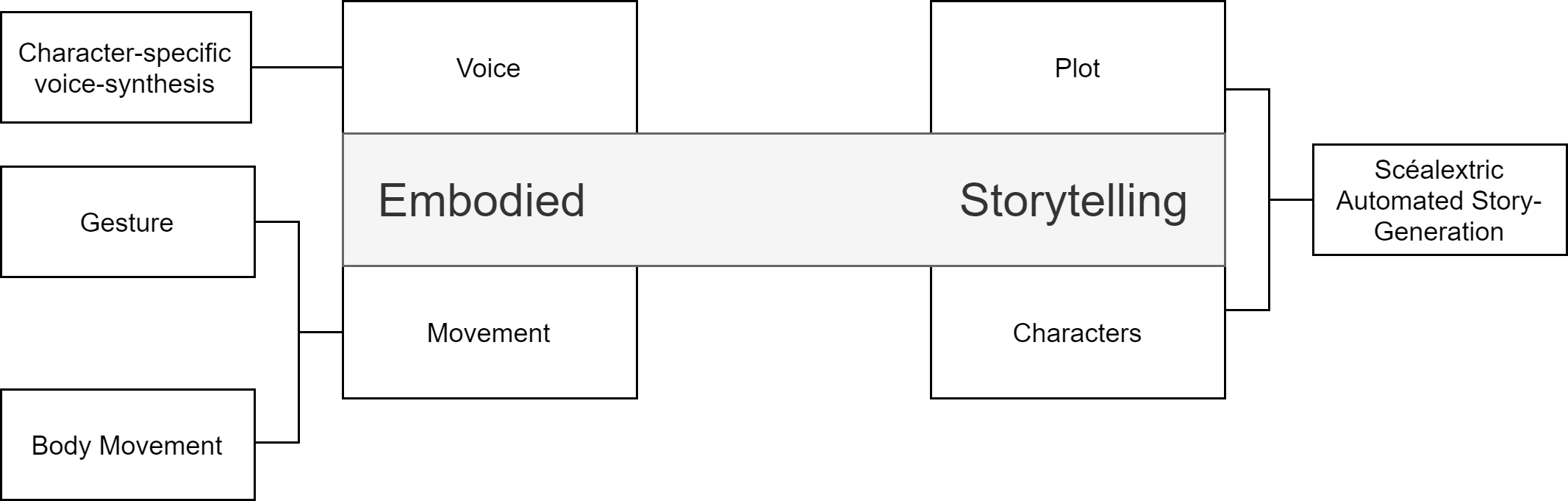

Supplement: Supplementary file 4 [file DataSheet1.ZIP › Frontiers_Creativity_Robotics_2020_Revised/model_01.png]

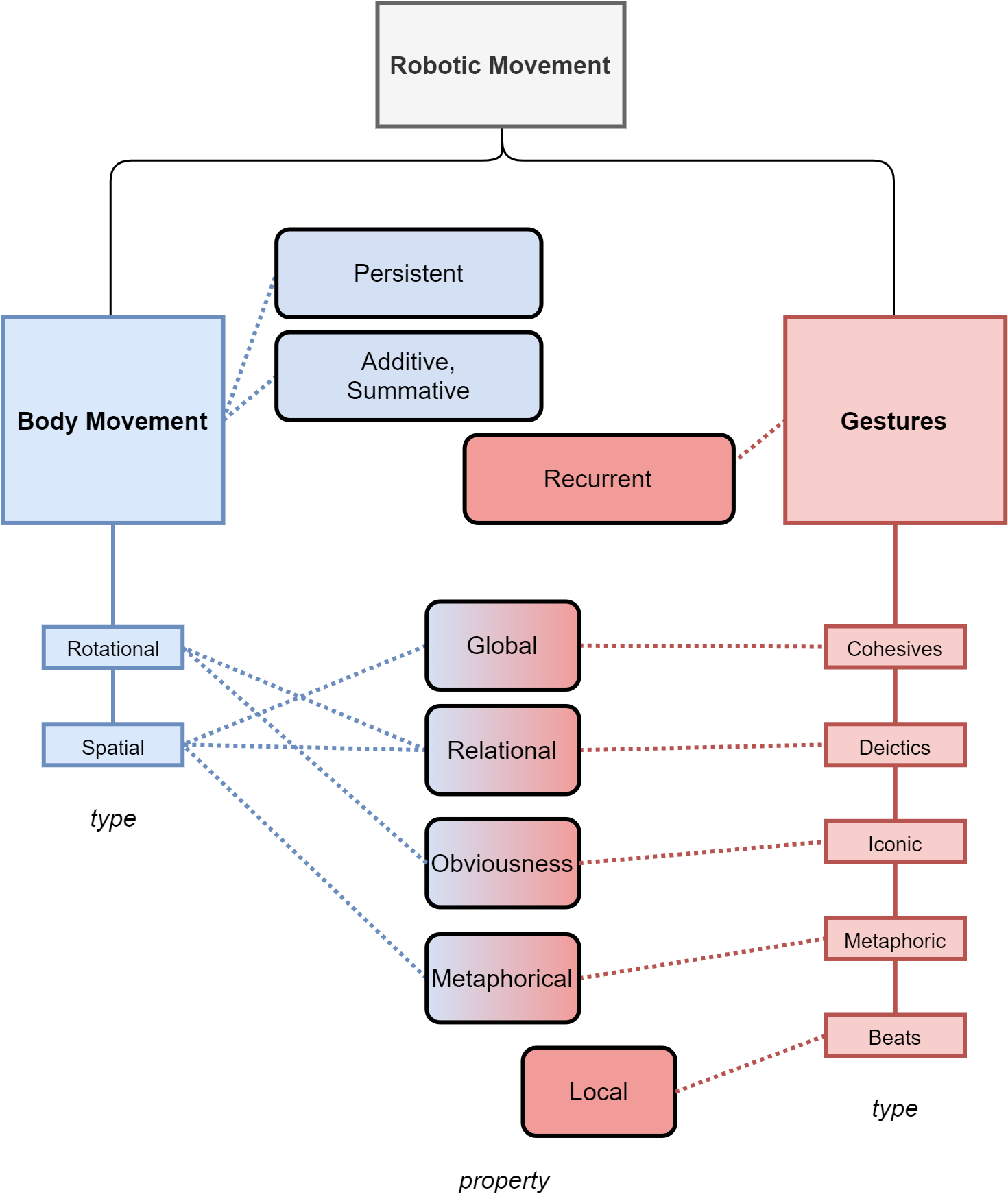

Supplement: Supplementary file 4 [file DataSheet1.ZIP › Frontiers_Creativity_Robotics_2020_Revised/model_03.png]

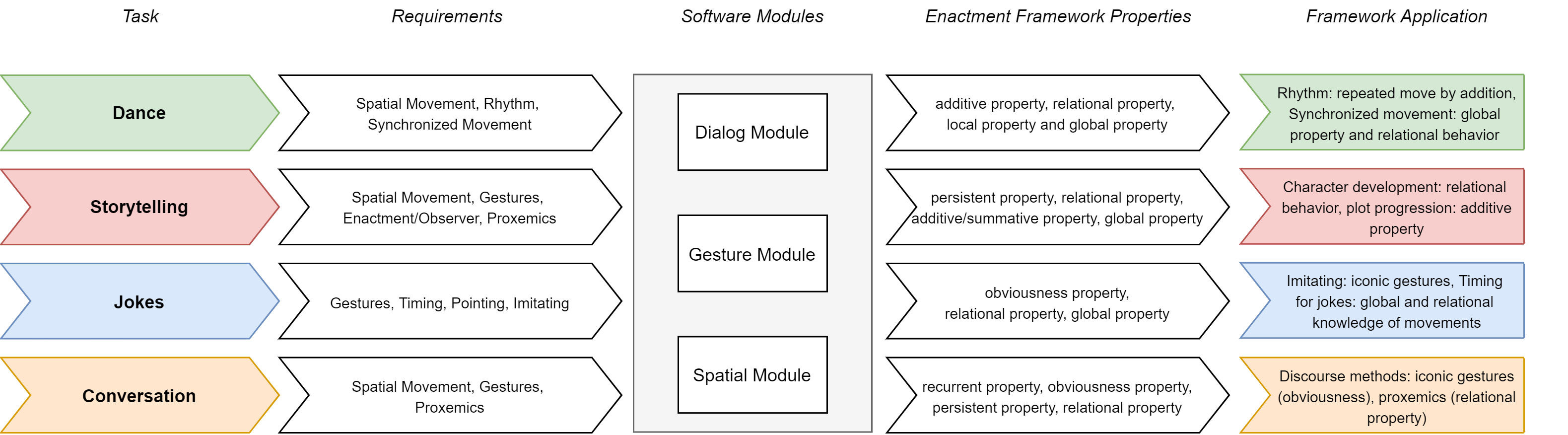

Supplement: Supplementary file 4 [file DataSheet1.ZIP › Frontiers_Creativity_Robotics_2020_Revised/model_04.png]

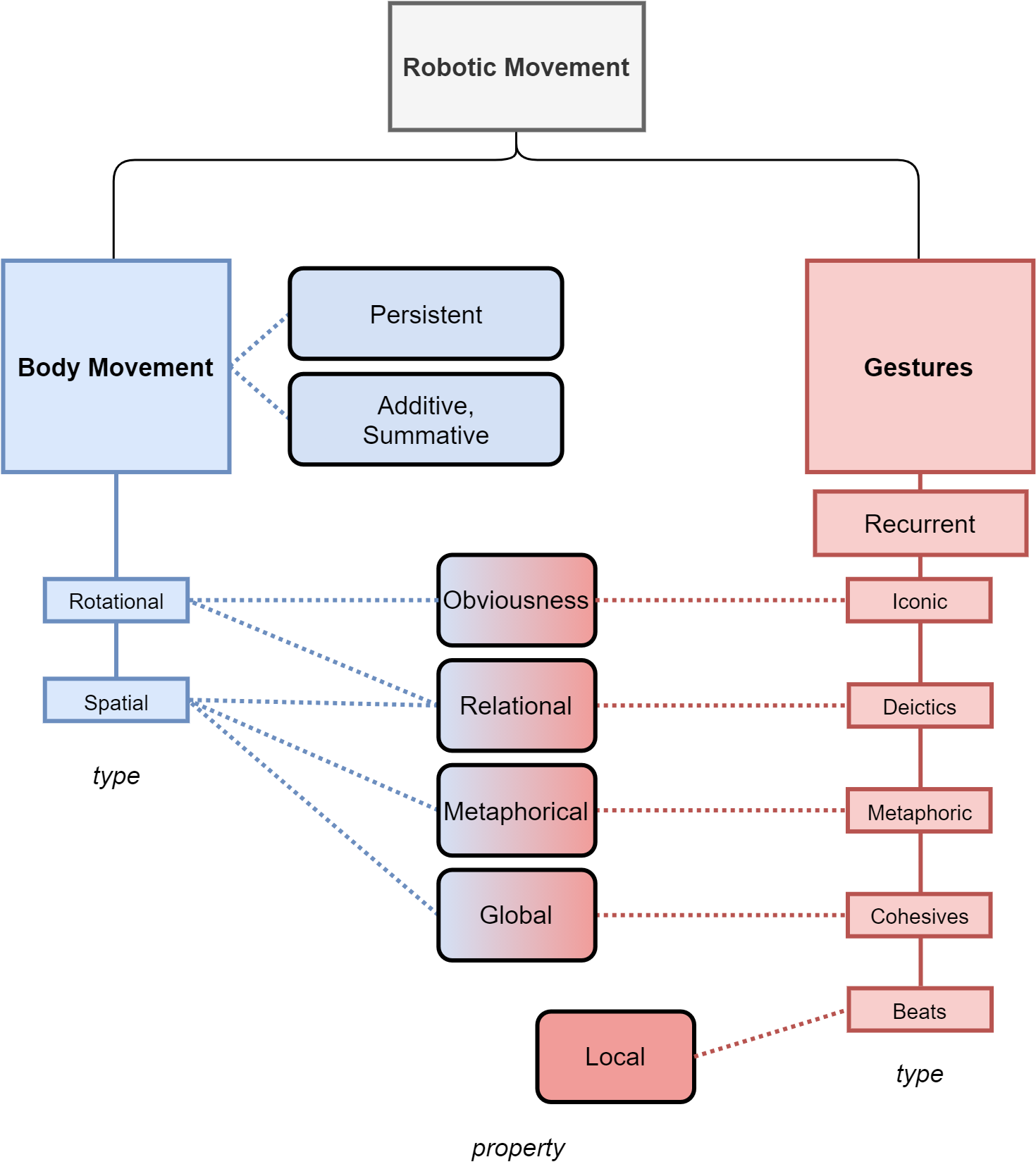

Supplement: Supplementary file 4 [file DataSheet1.ZIP › Frontiers_Creativity_Robotics_2020_Revised/model_06.png]

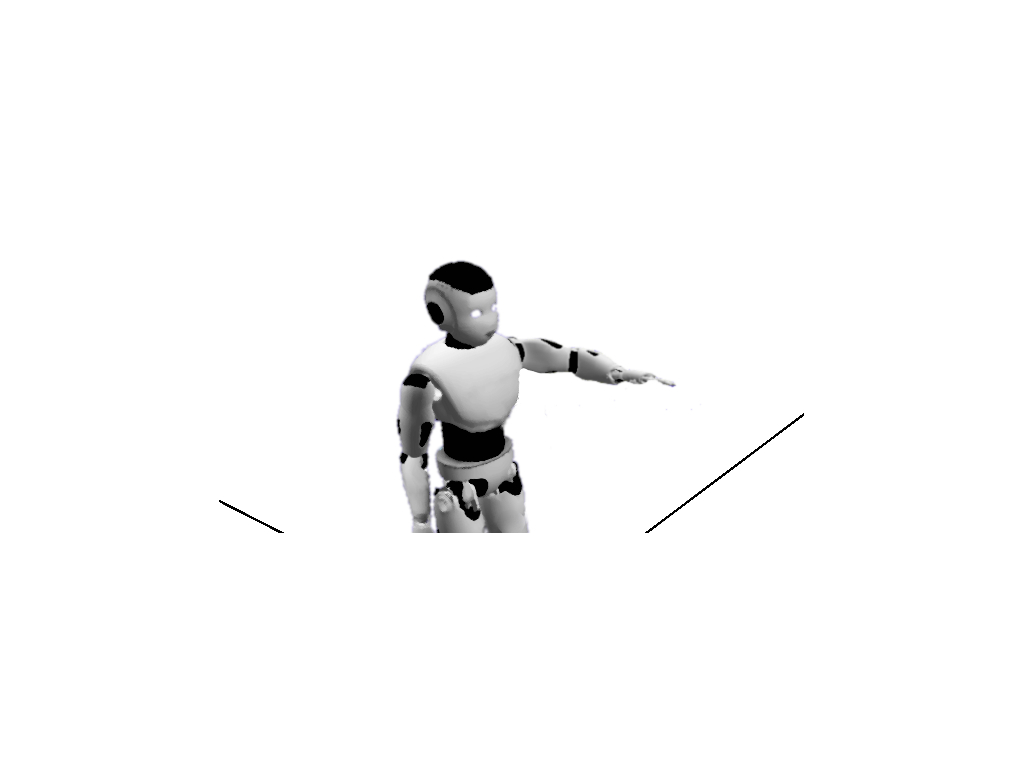

Supplement: Supplementary file 5 [file Image2.JPEG]

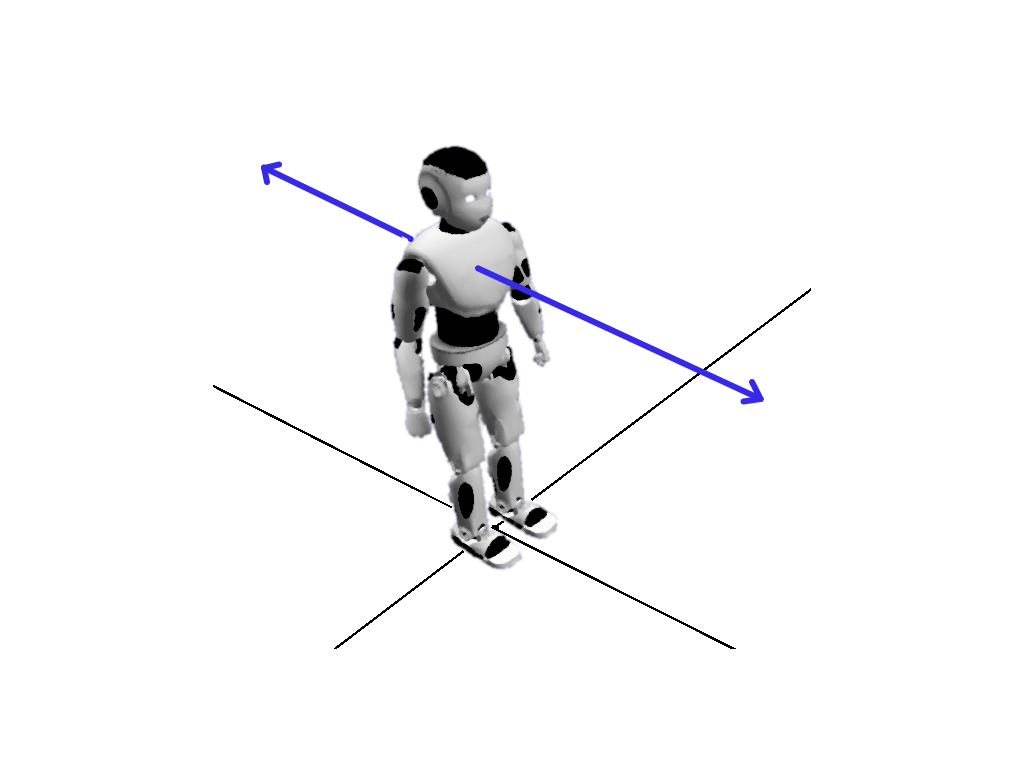

Supplement: Supplementary file 6 [file Image5.JPEG]

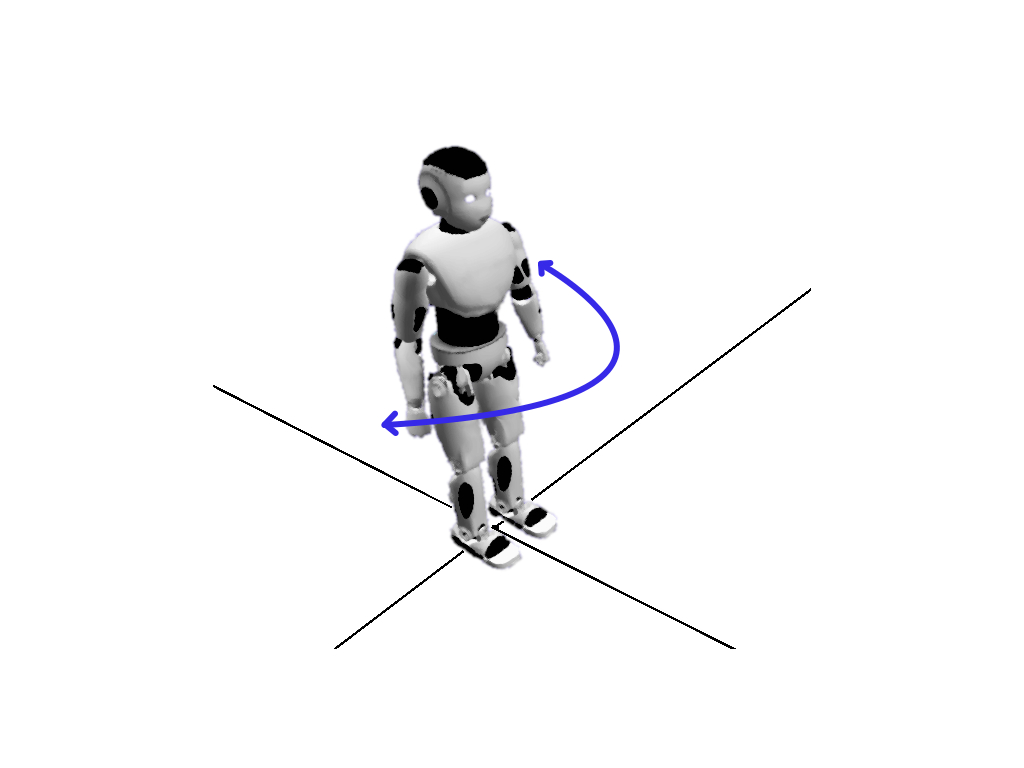

Supplement: Supplementary file 7 [file Image6.JPEG]
